# BALB/c mice challenged with SARS-CoV-2 B.1.351 β variant cause pathophysiological and neurological changes within the lungs and brains

**DOI:** 10.1099/jgv.0.002039

**Published:** 2024-10-30

**Authors:** Panatda Saenkham-Huntsinger, Aleksandra K. Drelich, Pinghan Huang, Bi-Hung Peng, Chien-Te K. Tseng

**Affiliations:** 1 Departments of Microbiology and Immunology, University of Texas Medical Branch, Galveston, TX, USA; 2 Neurobiology, University of Texas Medical Branch, Galveston, TX, USA

**Keywords:** B.1.351, BALB/c, neuro-COVID, neuropathogenesis, SARS-CoV-2

## Abstract

Up to one-third of individuals suffering from acute SARS-CoV-2 infection with the onset of severe-to-mild diseases could develop several symptoms of neurological disorders, which could last long after resolving the infection, known as neuro-COVID. Effective therapeutic treatments for neuro-COVID remain unavailable, in part, due to the absence of animal models for studying its underlying mechanisms and developing medical countermeasures against it. Here, we explored the impact of SARS-CoV-2 infection on the well-being of respiratory and neurological functions of BALB/c mice by using a clinical isolate of β-variant, i.e. B.1.351. We found that this β-variant of SARS-CoV-2 primarily infected the lungs, causing tissue damage, profound inflammatory responses, altered respiratory functions and transient but significant hypoxia. Although live progeny viruses could not be isolated, viral RNAs were detected across many anatomical regions of the brains in most challenged mice and triggered activation of genes encoding for NF*-kB*, *IL-6*, *IP-10* and *RANTES* and microglial cells. We noted that the significantly activated *IL-6*-encoded gene persisted at 4 weeks after infection. Together, these results suggest that this B.1.351/BALB/c model of SARS-CoV-2 infection warrants further studies to establish it as a desirable model for studies of neuropathogenesis and the development of effective therapeutics of neuro-COVID.

## Introduction

The COVID-19 pandemic has been declared ended since May 2023 by the World Health Organization. Despite the effective vaccination strategy over the past few years, especially against ancestral strains of SARS-CoV-2, many individuals continue suffering from infections by variants of concern (VOC) mutants that are known to escape to an alarming extent the adapted immunities acquired through natural infection and/or vaccination [1], resulting in the onset of post-acute sequelae of COVID (PASC), also known as long-COVID, that can persist for months or even years.

Among many symptoms associated with various tissues and organs in SARS-CoV-2-infected patients suffered from long-COVID, neurological manifestations, such as fatigue, loss of smell and taste, depression, headache, dizziness, anxiety, brain fog and attention ailment, are most recognized neurological disorders, also known as neuro-COVID [2,4]. While neuro-COVID occurred more frequently in individuals suffered from severe-to-moderate acute SARS-CoV-2 infection, it also happened in mild and even asymptomatic patients [4]. Studies with post-mortem brain specimens derived from confirmed COVID-19 patients have demonstrated the presence of viral RNA and/or viral particles, suggesting that this predominately respiratory infection of SARS-CoV-2 can spread to the brain/central nervous system (CNS) [5]. To access the CNS, SARS-CoV-2 may enter through peripheral nerves (e.g. olfactory nerve, trigeminal nerve or vagus nerve) or a haematogenous route via the disrupted blood–brain barrier (BBB) [6,8].

Despite the intense effort, the pathogenetic mechanism(s) of long- and/or neuro-COVID remains elusive largely due to the scarce of relevant brain specimens of confirmed COVID patients and the absence of SARS-CoV-2-permissive animal models that could develop key neurological symptoms mimicry of natural infection. However, several hypotheses implicate that neuro- or long-COVID could be induced directly upon SARS-CoV-2 infection of cellular components of the brain/CNS or indirectly upon exposure to the milieu of infection-triggered prolonged and exacerbated neuroinflammatory responses, causing activation of microglia. Furthermore, respiratory SARS-CoV-2 infection could activate platelets and coagulation, along with the formation of microvascular clots, which together can contribute to hypoxic neuronal injury and BBB dysfunction [9].

Mouse remains a popular choice of laboratory animal species to date for studies of viral pathogenesis and the development of effective medical countermeasures for human infectious diseases, largely due to its small size, relatively inexpensiveness and abundance of wide-spectrum of immunological reagents for studying host–pathogen interaction. Unfortunately, differences in the structure and genomic sequence of ACE2, the key functional receptor of SARS-CoV-2, between mouse and humans make the standard laboratory mice nonpermissive to ancestral strains of SARS-CoV-2, e.g. Wuhan and SARS-CoV-2 [US_WA-1/2020 isolates (WA-1)] that were widely spread among individuals during the early phase of COVID outbreak. Many efforts afforded by many laboratories with various genomic approaches, aiming at converting the non-permissiveness into permissiveness of mice to SARS-CoV-2 infection, have strived and succeeded in deriving many humanized mouse models, including transient ACE2-expressing Ad5-hACE2 mice, classic human keratin (K18) and CAG promoter-driven K18- and AC70-hACE2-transgenic (Tg) mice, respectively, and various knock-in (KI) versions of hACE2-Tg mice, that have been widely used and proven useful for studies of COVID-19 pathogenesis [10,14]. While various strains of hACE2 KI mice were proven susceptible to SARS-CoV-2 infection, infected mice failed to show any signs of illness, including weight loss, unless KI mouse-adapted (MA) virus exhibiting additional adaptation mutations was used, like the initial MA SARS-CoV-2/MA10 known to infect WT mice with the onset of disease and mortality in an age-dependent manner [15]. In contrast, classic K18- and AC70-hACE2-Tg mice were fully susceptible to clinical isolates of SARS-CoV-2, causing disease and mortality in a dose-dependent manner, in which the value of LD_50_ for the latter is merely 3 TCID_50_ of the SARS-CoV-2/WA-1 isolate [13]. While zoonotic SARS-CoV and MERS-CoV were largely considered respiratory pathogens, very few (if any) believed that these two highly pathogenic zoonotic human CoVs possessed any neurotropic potential to invade the brain/CNS, despite viral RNAs could be detected in the brains of up to one-third of patients who succumbed to infection, until the recently emerged SARS-CoV-2 [16,18]. Human CoVs are now generally recognized as neurotropic viruses and could negatively impact the brain health and overall well-being of individuals, as best exemplified by the frequent onset of neuro- or long-COVID in SARS-CoV-2-infected individuals [19]. While earlier studies using MA SARS-CoV-2/MA10 to infect BALB/c mice revealed the induction of acute lung injury and age-dependent mortality without any dateable signs of viral infection and pathology in the brain, suggesting that MA10 might not be neurotropic in nature [20,21], making it not ideal for studying the pathogenetic mechanism of neuro- or long-COVID. In the extreme opposite of the MA10/BALB model, the overwhelmed brain infection with Wuhan or WA-1 isolates of both K18- and AC70 hACE2-Tg mice, likely the main cause of mortality, similarly makes both classic ACE2-Tg mouse models less attractive for studying COVID-19 pathogenesis in humans. Although the initial clinical isolates of SARS-CoV-2 could not successfully infect WT mice, like ever-mutating RNA viruses, SARS-CoV-2 mutates frequently to overcome various factors that prevented the establishment of viral infection in mice and other potential hosts. To this end, many VOC strains of SARS-CoV-2 have been isolated; among which an isolate of b-variant of SARS-CoV-2, namely B.1.351 variant (TY8-612 strain), has the capacity to infect BALB/c mice without the need to adaptation, causing weight loss and pulmonary inflammatory responses [22]. However, whether this B.1.351 variant of SARS-CoV-2 could spread the infection from the respiratory tract to the brain of BALB/c mice, along with the potential to alter the biological functions of CNS, has not been fully explored.

In this study, we demonstrated that SARS-CoV-2 B.1.351 variant can readily infect BALB/c mice, resulting in the onset of severe lung pathology, impaired respiratory function and hypoxia, as evidenced by significant, but transient, reduction of saturated peripheral blood oxygen (SpO_2_). We also noted that infection-triggered inflammatory responses within the lungs were sustained even after the clearance of the infectious virus. Strikingly, we found that SARS-CoV-2-specific RNAs could be readily detected within the brain of most (if not all) infected mice, with the higher frequency detected at sites of the olfactory bulb (OB) and brainstem (BS), compared to the trigeminal nerve, cortex (CX) and cerebellum (CL), accompanied with the induction of inflammatory mediators. Together, our results indicate that SARS-CoV-2 B.1.351-infected BALB/c mice exhibited several pathophysiological outcomes as seen in confirmed patients suffering from varying symptoms of neuro-COVID, including the detectable SARS-CoV-2 RNA in the brain, hypoxia and prolonged inflammatory responses in both lung and brain. Together, we believe that this SARS-CoV-2 B.1.351/BALB/c model warrants further studies to better understand the likely impact of the lung–brain axis on the pathogenesis of neuro- or long-COVID.

## Methods

### Animals, tissue culture and virus

BALB/c mice were purchased from Inotiv (West Lafayette, IN). Vero E6 cells (ATCC, CRL:1586) were grown in minimum essential medium (MEM; Gibco) supplemented with 10% FBS, 100 units ml^−1^ penicillin and 100 µg ml^−1^ streptomycin. SARS-CoV-2 B.1.351 β variant (TVP23218, SA/ZA VAR) was propagated and quantified in Vero E6 cells by using Vero E6-based infectivity assays and expressed as logs TCID_50_ ml^−1^, as previously described [11], and stored at −80 °C until needed. The passage 2 (P2) of the virus stock was used for the challenge.

### Viral challenge

BALB/c mice (6–9 weeks old) were anaesthetized with isoflurane and intranasal (i.n.) challenged with 60 µl of 5×10^4^ TCID_50_ SARS-CoV-2 B.1.351 β variant (TVP23218, SA/ZA VAR) or 60 µl 2% FBS-MEM as mock control. Mice were monitored daily for mortality and morbidity. Morbidity is based on weight loss and clinical scores using 1–4 grading system as the following: 1: Healthy, 2: ruffled fur, lethargic, 3: hunched posture, orbital tightening, increased respiratory rate and/or >15% wt loss and 4: dyspnoea and/or cyanosis, reluctance to move when stimulated or >20% wt loss. Infected BALB/c mice were euthanized at 2, 4, 8 and 28 days post-infection (dpi). Lungs and brains were harvested for downstream experiments.

### Quantification of live virus titres in tissues harvested from infected mice

The frozen tissues (lungs or brains) were weighted and homogenized in PBS/2% FBS solution using the TissueLyser (Qiagen). Cellular debris was removed from homogenates by centrifugation. Cell debris-free homogenates were used to quantify infectious viruses in the standard Vero E6 cell-based infectivity assays in 96-well microtiter plates [11]. Briefly, tissue homogenates were tenfold serially diluted in 2% FBS–MEM media. Each diluted homogenate (100 µl) was added into Vero E6 cell plates and incubated at 37 °C with 5% CO_2_. Cytopathic effect (CPE) was read at 72 h after incubation, and the titre of the virus was expressed as 50% tissue culture infectious dose per gram of tissue (TCID_50_ g^−1^).

### RNA extraction and quantitative PCR for semi-quantification of viral RNAs

Tissues (lungs or brains) were weighted and homogenized in 1 ml of TRIzol reagent (Invitrogen) using TissueLyser (Qiagen). RNA was extracted using Direct-zol RNA miniprep kits (Zymo Research) by following the manufacturer’s instructions. Total RNA was treated with Turbo DNAse I (Invitrogen) followed by cDNA synthesis using iScript cDNA Synthesis kit (Biorad) according to the manufacturer’s instructions. Ten nanograms of cDNA were used as a template to amplify specific genes and viral RNA using specific primer sets (Table 1) and iQ SYBR green supermix (Biorad). The samples were run in duplicate using the CFX96 real-time system (Biorad) with the following conditions: 95 °C for 3 min, then 45 cycles of 95 °C for 15 s and 58 °C for 30 s. The level of expression was then normalized with 18S RNA and calculated using the 2^-∆∆Ct^ as a relative fold compared to the mock control animal.

**Table 1.** Oligonucleotide primer list

Primer name	Sequences (5'--> 3')
E gene SARS-CoV-2-F	ACA GGT ACG TTA ATA GTT AAT AGC GT
E gene SARS-CoV-2-R	ATA TTG CAG CAG TAC GCA CAC A
mIFN-ϒ-F	GCG GAC TTC AAG ATC CCT ATG
mIFN-ϒ-R	GCT GTT GCT GAA GAA GGT AGT A
mNF-kB1-F	GAA ATT CCT GAT CCA GAC AAA AAC
mNF-kB1-R	ATC ACT TCA ATG GCC TCT GTG TAG
mIL-6-F	CTG CAA GAG ACT TCC ATC CAG
mIL-6-R	AGT GGT ATA GAC AGG TCT GTT GG
mIP-10-F	ATC ATC CCT GCG AGC CTA TCC T
mIP-10-R	GAC CTT TTT TGG CTA AAC GCT TTC
mRANTES-F	TTT GCC TAC CTC TCC CTC G
mRANTES-R	CGA CTG CAA GAT TGG AGC ACT

### Histopathology, immunohistochemistry and immunofluorescence staining of infected tissues

Paraformaldehyde-fixed lung and brain tissues were paraffin-embedded for sectioning (5 µm) and haematoxylin and eosin (H and E) staining at the Histopathology Core Facility at University of Texas Medical Branch, Galveston (UTMB). Histopathology of lungs and brains of SARS-CoV-2-infected and mock-infected animals was evaluated by the designated pathologist at UTMB. For immunohistochemistry (IHC) and/or immunofluorescence (IF) staining, paraffin-embedded tissue sections were deparaffined and rehydrated by using the standard xylene-graded percentages of ethanol protocol, as previously described [11]. The antigens in the tissue sections were retrieved with the citrate buffer (pH 6.0) and pressure-cooked for 15 min, resulting in tissues on the slides being blocked with BloxAll and 2.5% normal serum. For IHC, slides were incubated with primary antibody, anti-SARS-CoV-2 nucleocapsid antibody (HL448) (1:5000, GeneTex; GTX635686), anti-Iba1 antibody (HL1880) (1:500, GeneTex; GTX637629), anti-CXCL4/PF-4 antibody (1:500 Sigma; SAB5701480) and anti-myeloperoxidase (MPO) (1:1000, Abcam; Ab208670) at room temperature for 1 h followed by incubating with the second antibody, ImmPress HRP Horse anti-rabbit IgG Polymer kit (MP-7401), then revelated using Immpact DAB EqV substrate kit (Vector Laboratories, SK-4103) and counterstained with haematoxylin solution (Sigma, MHS16). For IF, slides were incubated with primary antibody, anti-GFPA (1:500, GeneTex: GTX85454) overnight at 4 °C. Next, slides were incubated with secondary antibody (1:1000, AlexaFlour 488 goat anti-chicken IgG) and imaged under the digital microscope (OlymPus DP71).

### Mouse respiratory function and saturated blood oxygen (SpO_2_) analysis

The baseline of respiratory function and SpO_2_ of BALB/c mice was monitored 1 day prior to the infection by using mouse whole-body plethysmography (WBP, DSI™) and MouseOx^R^ Plus (STARR™ Life Sciences Corp.), respectively. Virally challenged mice were similarly monitored for their prospective respiratory function and the content of SpO_2_ daily, starting from 1 to 8 dpi. The respiratory function parameters, Penh, EF50 and Rpef, and SpO_2_ were presented as the mean values of ten mice.

### Flow cytometry

The half-sphere brain was homogenized in 5 ml R10 medium [RPMI 1640 (Gibco, 22400089) supplemented with 10% FBS and 1% penicillin–streptomycin] using a 7 ml Dounce tissue grinder. The homogenate was then centrifuged at 300 ***g*** for 10 min at 4 °C. The cells were resuspended in 5 ml of 37% Percoll solution (Cytiva, 17089102), transferred on the top of the 70% Percoll solution and centrifuged at 800 ***g*** for 1 h at 23 °C without break to separate the myelin and lymphocytes. The layer containing lymphocytes was transferred into a new tube, and 3 volumes of 1× HBSS (Gibco, 14025126) were added and centrifuged at 300 ***g*** for 10 min. Cells were then resuspended in 40 µl of FACS buffer (PBS supplemented with 2% of FBS) containing fluorochrome-conjugated surface staining antibodies and incubated for 1 h at 4 °C, followed by cell fixation using Cytofix/Cytoperm buffer (BD Bioscience, 555028) for 20 min at 4 °C. The cells were washed twice with 1× PBS and resuspended in 300 µl FACS buffer for analysis. The fluorochrome-conjugated anti-mouse antibodies included FITC-conjugated CD3 (17A2, Biolegend), efluor450-conjugated CD4 (GK1.5, eBioscience), APC-eFluor780-conjugated CD8 (53–6.7, eBioscience), APC-conjugated CD44 (IM7, Biolegend), PE-Cyanine7-conjugated CD62L (MEL-14, Biolegend), PE-Dazzle594-conjugated CD69 (H1.2F3, Biolegend) and PerCP-eFluor710-conjugated CD103 (2E7, eBioscience). Fixable Viability Dye eFluor 506 (eBioscience, 65-0866-14) was also used in the call staining. Cells were analysed on BD LSRFortessa and using Flowjo v10 for further analysis.

### Statistical analysis

Statistical analyses were performed using GraphPad Prism software. Values from multiple experiments are expressed as means±sem. Statistical significance was determined for multiple comparisons using one-way ANOVA. Student’s t-test was used for comparisons of two groups. Results were regarded as significant if two-tailed *P* values were <0.05. All data are expressed as mean±sem.

## Results

### Kinetics of SARS-CoV-2 B.1.351 β variant infection in the lungs and the subsequent host responses of BALB/c

**Fig. 1.**
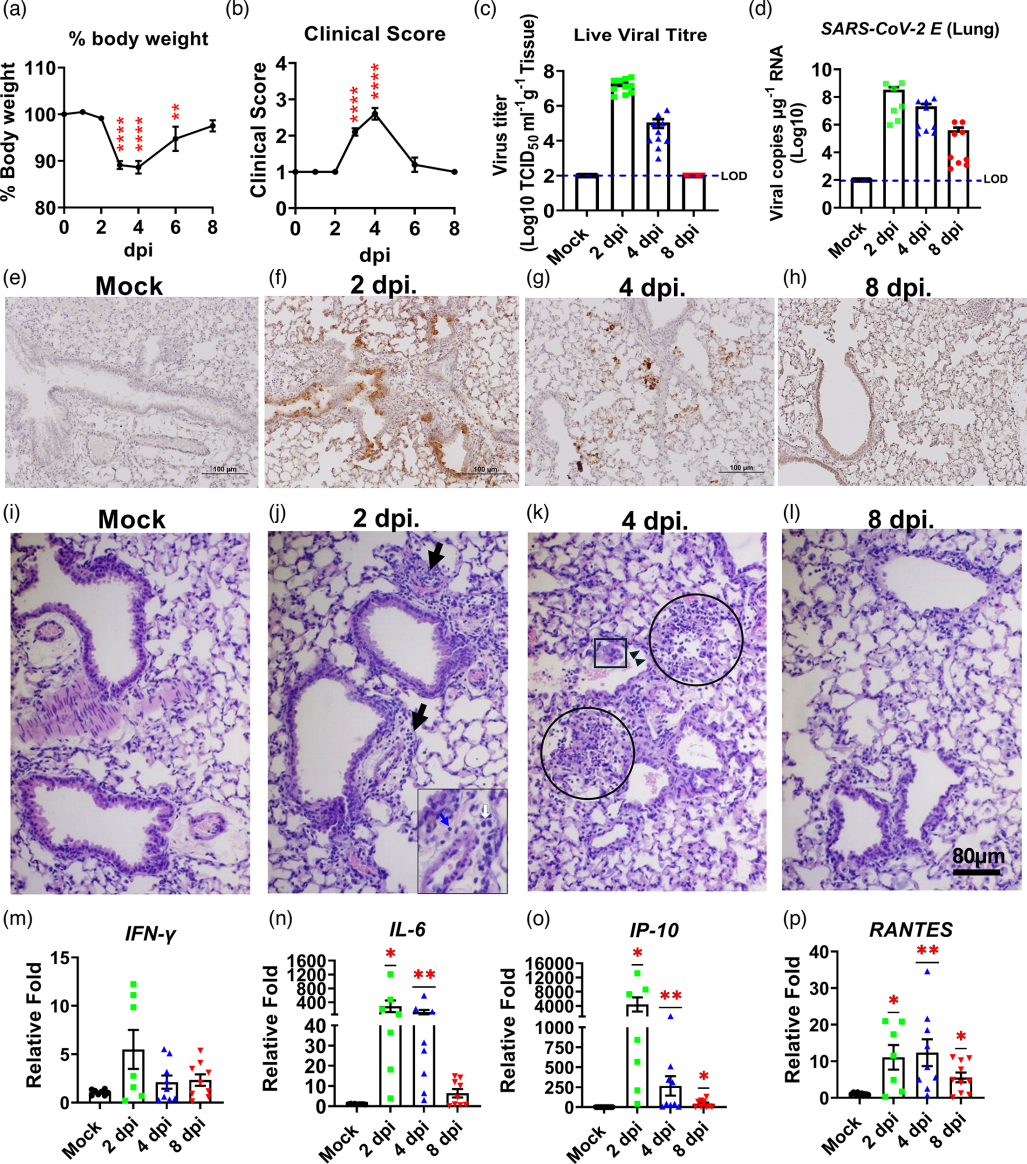
BALB/c mice challenged with B.1.351, a β variant of SARS-CoV-2, exhibited weight loss and other signs of illness, along with pulmonary immunohistopathology. BALB/c mice were i.n. challenged with SARS-CoV-2 B.1.351 clinical isolate, and the transient weight (**a**) and other clinical signs (**b**) were noted over the course of infection of 8 days. Virus progeny (**c**) and RNA (**d**) in the lungs of SARS-CV-2-infected and mock-infected mice were determined by Vero-E6-based assay and qPCR (*n*=10), respectively. The dotted line represents the limit of detection (LOD). The detection of SARS-CoV-2 N-protein was done by IHC in mock (**e**), 2 dpi (**f**), 4 dpi (**g**) and 8 dpi (**h**) after the infection. Representative of the H and E image showed histopathology of the lungs from mock (**i**), 2 dpi (**j**) with inflammatory infiltrations (black arrows), mainly lymphocytes (blue arrow in the inset) and monocytes/macrophages (white arrow in the inset), 4 dpi (**k**) number of inflammatory cells increased, migrated to the surrounding area (circle) and caused respiratory epithelial damage, detachment of epithelia (square) from smooth muscle layer (arrowhead) and the inflammation has subsided by 8 dpi (**l**). The transcription level of *IFN-ɣ* (**m**), *IL-6* (**n**), *IP-10* (**o**) and *RANTES* (**p**) was examined by qPCR (*n*=7–10). Data are expressed as mean±sem, *P<0.05, and **P<0.01 as determined by the one-sample Wilcoxon test.

WT BALB/c mice were intranasally challenged with 5×10^4^ TCID_50_ of SARS-CoV-2 B.1.351 β variant. Mice were observed daily for signs of morbidity and mortality (if any) for up to 8 dpi. While no mortality was observed, infected mice started to lose weight on 2 dpi, reaching significant weight losses and other signs of illness at 3 and 4 dpi, and gradually recovered afterwards (Fig. 1a, b). The virus yield in the lung tissues was determined by using the standard Vero E6-based infectivity assay and quantitative PCR (qPCR targeting the E gene of the virus at 2, 4 and 8 dpi. The peak levels of the infectious progeny virus and viral RNA copies were detected at 2 dpi with 7.26 log_10_ TCID_50_ g^−1^ of tissue and 8.68 log_10_ copies µg^−1^ of total RNA, respectively. Even though the live virus titres were declined to 5.05 log_10_ TCID_50_ g^−1^ of tissue and below the detection limit at 4 and 8 dpi, respectively, virus RNA persisted up to 8 dpi (5.60 log_10_ copies per µg RNA) when the study was terminated (Fig. 1c, d). Consistent with the readily detectable live viruses, positive IHC staining using antibodies specifically against SARS-CoV-2 nucleocapsid (N) protein was revealed within the lungs of challenged mice at both 2 and 4, but not at 8 dpi (Fig. 1e–h). Histopathology study by H and E staining showed detectable inflammatory infiltrates, mainly composed of lymphocytes and monocytes/macrophages, around bronchial/bronchiolar and perivascular areas of the lungs at 2 dpi (Fig. 1j) when compared to mock-infected mice in which few surveillant white blood cells can be detected (Fig. 1i). Such cellular infiltrates intensified and expanded to other areas at 4 dpi, accompanied with damaged respiratory epithelial cells (Fig. 1k). Despite the undetectable live virus, cellular infiltration persisted up to 8 dpi (Fig. 1l), albeit to a lesser intensity than those of 2 and 4 dpi. The lymphoid aggregates surrounded by plasma cells were observed in 80% (four out of five mice) of infected mice at 28 dpi (Fig. S1, available in the online version of this article). Additionally, we noted that the transcriptional expressions of genes encoding for *INF-ɣ*, *IL-6*, *IP-10* and *RANTES* were induced at varying intensities in the lungs at 2 dpi and declined at 4 and 8 dpi (Fig. 1m–p). However, the induction of *IL-6*, *IP-10* and *RANTES* transcripts remained significant at 4 and 8 dpi when compared to mock-challenged mice (Fig. 1m–p).

### Respiratory challenge with B.1.351 β variant of SARS-CoV-2-activated platelets and neutrophils

**Fig. 2.**
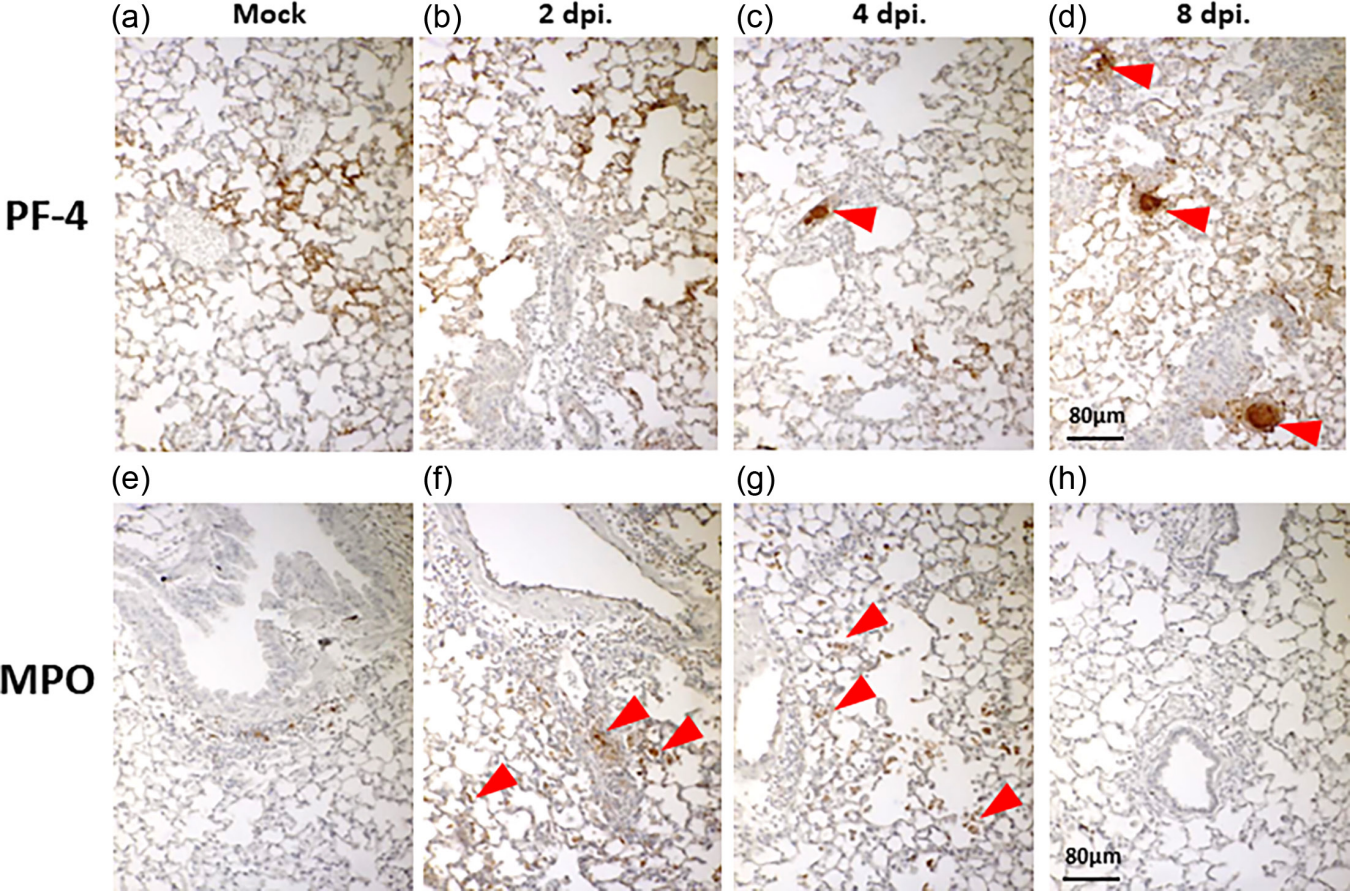
Respiratory infection with β variant of SARS-CoV-2, B.1.351, resulted in activation of platelets and neutrophils within the lungs of BALB/c mice. Platelet activation was assessed using platelet factor 4 (PF-4) as the primary antibody. Capillaries of uninfected lungs showed signs of activated platelets (**a**). However, the intensity and area of activated platelets increase at 2 dpi (**b**). At 4 dpi (**c**), few arterioles showed signs of platelet aggregates (arrow) and the number of such aggregates detectable in arterioles (arrow) increased at 8 dpi (**d**). Neutrophilic activation was assessed using antibody to MPO as the primary antibody. Numbers of activated neutrophils (brown colour) increased in perivascular regions at 2 dpi (f, arrows) and gradually migrated along with macrophages to alveolar space at 4 dpi (g, arrows). By 8 dpi (h), most of the activated neutrophils were no longer detectable. However, few activated neutrophils were detected in uninfected mouse as well (**e**). Bar=80 µm.

The interplay between inflammation, especially activated neutrophils, and platelets is often observed in SARS-CoV-2 infection and associated with a significant risk of immune-thrombotic complication [23,24]. As cellular infiltrates and transcriptional expression of several inflammatory mediators were detected within the lungs of B.1.351 β-infected mice (Fig. 1i–p), we performed IHC staining against platelet factor-4 (PF-4) and MPO, markers of platelet and neutrophil/neutrophil extracellular trap (NET) activation, respectively. To this end, we noted that highly activated platelets that manifested as elevated immunoreactivity at capillaries (Fig. 2b) could be observed at 2 dpi compared to the lungs of mock-infected mice, in which only a few small foci could be observed (Fig. 2a). Few arterioles displayed immunoreactivity with platelet aggregates that could be observed at 4 dpi. Interestingly, the number of immunoreactive arterioles increased at 8 dpi and persisted up to 28 dpi (Figs 2c, d and S2). Like the activated platelets, we found that the number of activated neutrophils was increased at 2 dpi, especially in the perivascular region (Fig. 2f) and migrated to alveolar apace closely associated with macrophages at 4 dpi, but not at 8 dpi (Fig. 2g, h). In contrast, we could only observe a few immune cells in the peribranchial region of mock-infected mice (Fig. 2e). These results indicate that B.1.351 β infection could activate both neutrophils and platelets, along with aggregated platelets within arterioles of BALB/c mice.

### B.1.351 β variant infection resulted in impaired respiration and transient but significantly reduced oxygen levels in the blood

**Fig. 3.**
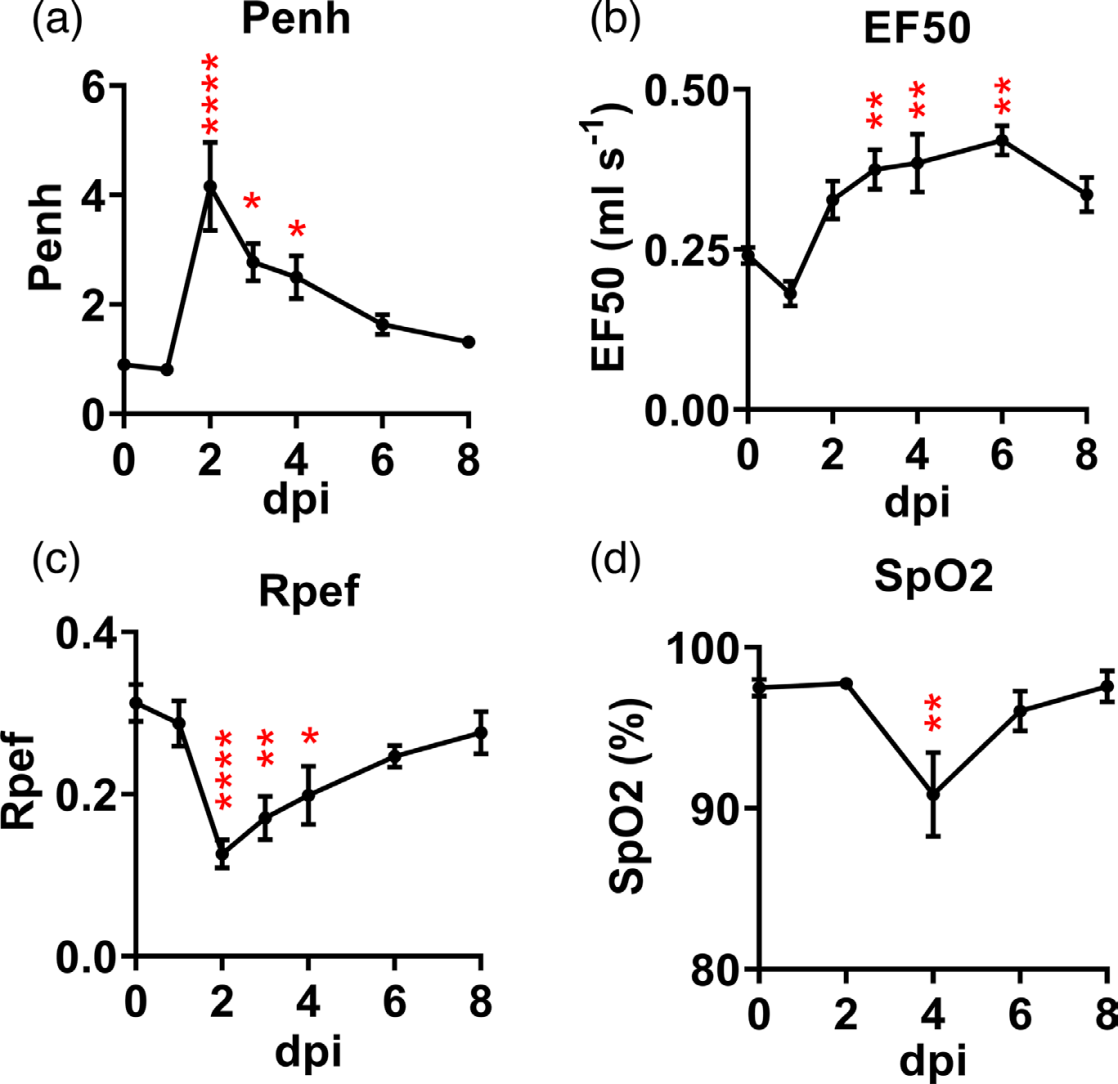
SARS-CoV-2 B.1.351 β variant infection impaired the respiratory function and caused transient SpO_2_ reduction in BALB/c mice. Respiratory function and SpO_2_ of SARS-CoV-2 B.1.351-infected BALB/c mice were measured daily from 1 to 8 dpi compared to the day before infection. Penh (**a**), EF50 (**b**), RpeF (**c**) and SpO_2_ (**d**). *N*=10, Data are expressed as mean±sem, *P<0.05, **P<0.01, ***P<0.001 and ****P<0.0001 as determined by one-way ANOVA.

Damaged pulmonary epithelial cells and the inflammatory responses elicited by B.1.351 β variant-infected mice, as shown in Fig. 1i–p, prompted us to investigate if the respiratory function could be altered as well. Thus, several parameters of the respiratory function of newly infected mice were monitored daily by using mouse WBP. The results were compared to the baseline measurements taken on the day prior to the infection. We noted that infection by this β-variant impaired the respiratory functions, as evidenced by an increase in the enhanced pause (Penh) and midexpiratory flow (EF50), accompanied by decreases in the peak expiratory flow rate (Rpef) as shown in Fig. 3a–c, respectively. The severity of distressed respiratory functions reached the peak at 2 dpi, gradually improved thereafter and returned to the baseline levels measured before infection. This pattern of respiratory functions was consistent with that of the morbidity (weight changes and other signs of illness) and the kinetics of viral infection in the lungs. Using the same cohort of mice, we also measured the level of SpO_2_. We found that SpO_2_ was significantly decreased from 97.5±2.1% in uninfected mice and 90.9±2.1% in infected mice at 4 dpi (Fig. 3d).

### Activation of transcription level of selected inflammatory mediators with detectable virus RNA in the brain of SARS-CoV-2 β variant (B.1.351)-infected BALB/c mice

**Fig. 4.**
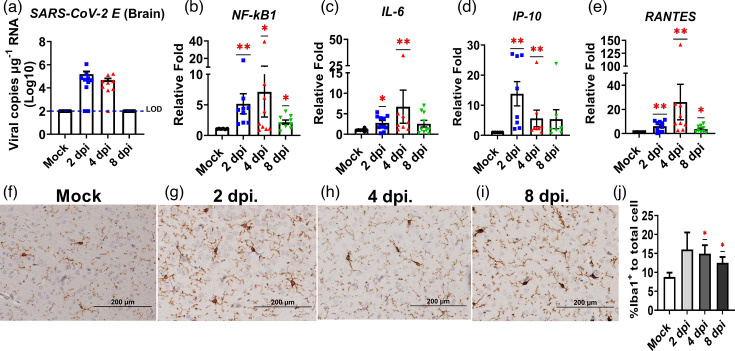
Respiratory challenges of BALB/c mice infected with B.1.351, a β variant SARS-CoV-2, resulted in readily detectable viral RNAs and promoted microglial activation and inflammatory secretion within the brains. SARS-CoV-2 RNA (E-gene) in the brains of infected BALB/c mice and mock control mice was detected by using qPCR (**a**, *n*=10). The dotted line is an LOD. Brain pro-inflammatory cytokine and chemokine expression level was determined by qPCR (*n*=7–10), *NF-kB* (**b**), *IL-6* (**c**), *IP-10* (**d**) and *RANTES* (**e**). Data are expressed as mean±sem, *P<0.05 and **P<0.01 as determined by the one-sample Wilcoxon test. Microglia activation was determined by IHC staining against Iba1 antibody at 2 dpi (**g**), 4 dpi (**h**), 8 dpi (**i**) compared with (**f**) mock-infected mouse brains. (**j**) Calculated percentage of Iba1+ cell to total cells of ten random Iba1-IHC images by ImageJ. Data are expressed as mean±sem, *P<0.05 as determined by the one-sample t-test. Iba1-IHC images are representative images from three animals per time point.

As infectious progeny virus could not be recovered in the brain of BALB/c mice challenged with MA SARS-CoV-2, named MA10 [21], it was assumed that the MA10 virus was not neuroinvasive with the infection largely restricted within the lungs. To investigate whether this β-variant, like MA10, is not neuroinvasive as well, we determined the yields of live virus and viral RNAs in the brains of B.1.351-infected BALB/c mice. Consistent with the earlier studies with the MA10 virus, we were unable to detect infectious virus over time from the brains. However, to our surprise, low but readily detectable viral RNAs could be identified within the brains, i.e. 5.20 and 4.67 log_10_ copies µg^−1^ RNA, at 2 and 4 dpi, respectively, and were no longer detectable at 8 dpi (Fig. 4a), suggesting that a low level of infection, relative to the lung infection, might have occurred in the brain. To determine whether such a less prominent B.1.351 infection could trigger host responses in the brain, we first examined whether microglial cells were activated by performing the standard IHC staining using specific antibodies against Iba-1, a marker of microglial cells. We demonstrated microglial cells were indeed activated at 2, 4 and 8 dpi, as compared to those of mock-infected mice. (Fig. 4f–j). Next, we assessed the inflammatory responses in the brain to semi-quantify the transcripts of genes encoding inflammatory mediators by using qPCR. We found that transcriptional expression of *NF-kB-*, *IL-6-* and *RANTES-*encoded genes were significantly induced as early as 2 dpi, with the peak levels of expression occurring at 4 dpi and subsided at 8 dpi. (Fig. 4b, c, e). We also found that the expression of *IP-10* was significantly increased at both 2 and 4 dpi, with the highest level detected at 2 dpi. (Fig. 4d). However, no alterations in brain histopathology were observed in the infected mice when compared to the mock-infected mice (data not shown). Together, the results suggested that respiratory β variant infection could activate microglia and induce the inflammatory response within the brains of BALB/c mice.

### Detection of viral RNA and various cytokines in various anatomy regions of the brain of infected BALB/c mice

**Fig. 5.**
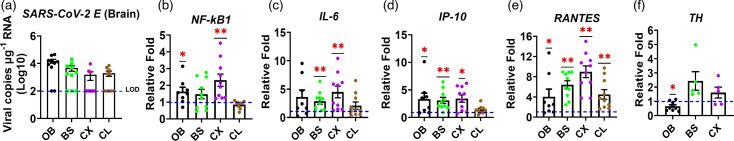
SARS-CoV-2 virus RNA distribution and cytokine profiling within various anatomic regions of the brains derived from BALB/c mice challenged with B.1.351, a β variant of SARS-CoV-2. BALB/c mice were infected with SARS-CoV-2 B.1.351 β variant, and brains were harvested and micro-dissected at 4 dpi. (**a**) Virus RNA was determined by qPCR at various brain regions (*n*=10). The dotted line is an LOD. The transcription level of *NF-kB* (**b**), *IL-6* (**c**), *IP-10* (**d**), *RANTES* (**e**) and *TH* (**f**) was examined at various brain regions (*n*=7–10). The relative fold was compared with the expression in mock control samples (blue-dotted line). Data are expressed as mean±sem, *P<0.05 and **P<0.01 as determined by the one-sample Wilcoxon test.

It has been shown that viral RNAs and proteins could be located at brain regions of confirmed COVID-19 patients [9]. As we were able to detect viral RNA in the brains of a great majority (i.e. 89%) of infected mice at 4 dpi (Fig. 4a), we investigated the anatomic distribution of viral RNA in the infected brain at 4 dpi. Specifically, the newly infected and mock-infected BALB/c mice were euthanized at 4 dpi and different brain regions, including Olfactory bulb (OB), Cortex (CX), Brainstem (BS) and Cerebellum (CL), were micro-dissected and subjected to total RNA extraction for qPCR analyses to quantify SARS-CoV-2 E gene expression. Among different brain regions, we noted that the highest amount of viral RNA copies (i.e. 4.18 Log_10_ copies per µg RNA) was detected in OB with high frequency (80%, eight out of ten mice) (Fig. 5a). The virus RNA was also found in BS (70%, seven out of ten mice), CL (50%, five out of ten mice) and CX (30%, three out of five mice) with 3.68, 3.29 and 3.18 Log_10_ copies per µg RNA, respectively (Fig. 5a). The transcriptional expression of genes encoding *NF-kB*, *IL-6*, *IP-10* and *RANTES* were measured in different brain regions as well. We found that only at CX region showed a significant increase in all the tested inflammatory mediator expression levels, while the levels of *IL-6*, *IP-10* and *RANTES* expression were significantly increased within in BS of infected mice compared to mock control (Fig. 5b–e). However, *RANTES* was the only inflammatory mediator that increased in CL. Additionally, we observed significantly elevated expressions of *IP-10* and *RANTES* within OB (Fig. 5d, e). Together, these results indicated that the distribution of viral RNAs and the inflammatory responses were not directly correlated throughout the brain, suggesting that the impact of SARS-CoV-2 infection on brain dysfunction might be largely dependent on the pathological site within the brain.

It has been well established that OB contains mostly dopaminergic (DA) neurons and can be recognized by the expression of tyrosine hydroxylase (TH), a rate-limiting enzyme of the catecholaminergic pathway [25,26]. As OB derived from infected mice harboured the highest levels of viral RNAs (Fig. 5a), we investigated whether B.1.351 infection could alter the expression of TH within OB and other brain regions. qPCR was used to determine the expression of *TH* gene in OB, BS and CX. We found the transcriptional expression of the TH-encoded gene was downregulated exclusively with OB, but not BS and CX, of infected mice, compared to mock-infected mice (Fig. 5f).

### Upregulated transcriptional IL-6 expression was sustained within the brains of B.1.351 β-infected BALB/c mice at 4 weeks, along with increased resident T cells

**Fig. 6.**
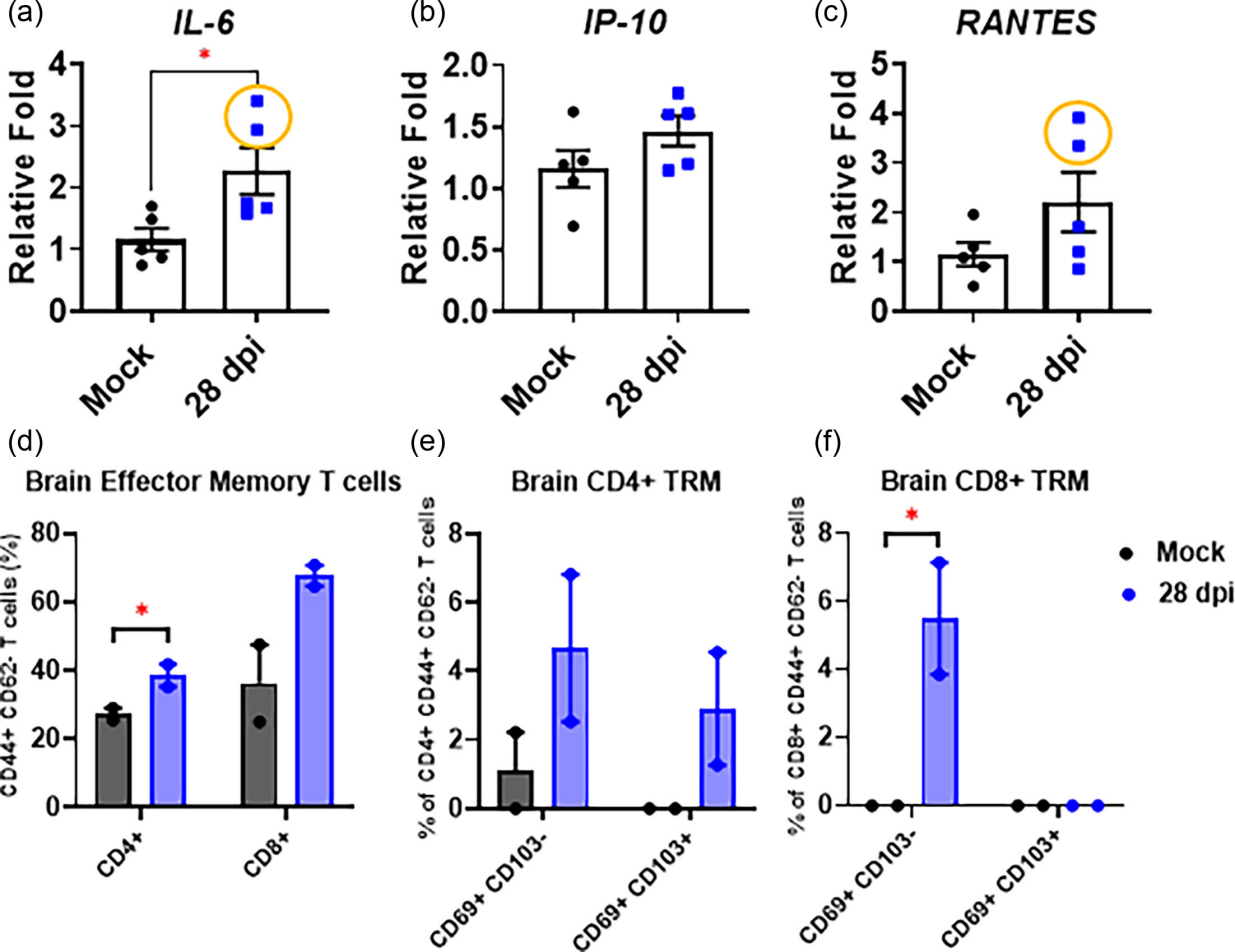
The prolonged induction of IL-6 and increase in resident memory T cells within the brains of BALB/c mice at 28 days after challenged with B.1.351 β variant of SARS-CoV-2. The transcription expression of gene encoding *IL-6* (**a**), *IP-10* (**b**) and *RANTES* (**c**) was done by qPCR from the total RNAs extracted from the brains of infected and uninfected BALB/c mice (*n*=5) at 28 dpi after the infection. The brain effector memory T cells (**d**), brain resident memory CD4+ (**e**) and CD8+ (**f**) T cells were determined in two infected mouse brains (with high levels of IL-6 and RANTES, orange circle) compared to the mock-infected brains at 28 dpi. Data are expressed as mean±sem, *P<0.05 as determined by the Mann–Whitney test.

To investigate whether the SARS-CoV-2 B.1.351 β variant infection could alter the brain immune responses in BALB/c mice, transcripts of genes encoding IL-6, IP-10 and RANTES were semi-quantified by qRT-PCR at 4 weeks post-infection. Compared to that of mock-infected controls, we found that transcriptional expression of the IL-6-encoded gene remained significantly elevated, whereas those of IP-10 and RANTES were no longer significantly elevated (Fig. 6a–c). Additionally, no microglia activation was observed at 28 dpi after the infection (Fig. S3). However, it was noted that two mice showing elevated IL-6 expression also had increased RANTES expression (Fig. 6a, c with circle). Such a sustained expression of both IL-6 and RANTES of these two infected mice prompted us to investigate the status of resident memory T cells within the brains. Briefly, single cells were isolated from half of the brain hemisphere and subjected to staining for surface markers specific for effector memory T cells and tissue-resident T cells. Results derived from flow cytometry indicated that the populations of effector memory T cells, i.e. CD44^+^ CD62^-^, were increased in both mice at 4 weeks post-B.1.351 β infection when compared to the mock-infected controls (Fig. 6d). We also investigated the expression of CD69, a marker for tissue-resident T cells, abbreviated as TRM, along with surface CD103 expression on brain T cells. We showed that CD4^+^ TRMs (i.e. CD69^+^ CD103^-^ and CD69^+^ CD103^+^) were increased in the infected mice as well, when compared with that of mock-infected controls. However, we were only able to detect CD69^+^CD103^-^, but not CD69^+^CD103^+^, CD8^+^ TRMs in infected mice (Fig. 6e, f). Together, the results suggested that B.1.351 β infection resulted in sustained activation of IL-6-encoded gene at least up to 4 weeks, which might promote retention of resident memory T cells in the absence of readily detectable cellular infiltrates within the brains.

## Discussion

Understanding the neuro-pathogenetic mechanism of SARS-CoV-2 is an urgent need for the development of therapeutics for long-COVID and neuro-COVID. However, it remains a huge challenge to establish suitable animal models for SARS-CoV-2 with the onset of at least some clinical manifestations mimicry of long- and/or neuro-COVID in patients. In this study, we characterized the pathophysiological outcomes in the lung and brain of the standard WT BALB/c mice that are naturally permissive to a β-variant of SARS-CoV-2 (i.e. B.1.351 isolate) without prior adaptation in mice.

We found that B.1.351-infected BALB/c mice exhibited an onset of acute and productive infection with the peak level of live progeny virus recovered at 2 dpi concomitant with intense inflammatory responses in the lungs, the major target of viral infection. Infected mice also showed transient but significant weight loss and other signs of illness between 3 and 6 dpi, with the maximum weight loss observed at 4 dpi. Infected mice regained the weight from 4 dpi and onwards with 100% recovery by 8 dpi. Additionally, we also noted elevated transcriptional expressions of genes encoding for IFN-ɣ, IL6, IP-10 and RANTES in lungs harvested from infected animals with the peak at 2 dpi (Fig. 1). Dysregulated and often exacerbated inflammatory responses, often referred as ‘cytokine storm’, triggered by SARS-CoV-2 infection have been referred as a hallmark of COVID-19 [27], making the pronounced inflammatory activation of infected mice consistent with patients. Importantly, we noted elevated activation of selected inflammatory genes, with the IFN-ɣ-encoded gene as the only exception, sustained at the time when the live virus was no longer detectable. Yasui *et al* [24] have revealed that infection of the SARS-CoV-2 B.1.351 variant caused lethality in BALB/c mice of ~6-month-old with a massive and sustained production of multiple pro-inflammatory cytokines and chemokines. Like mouse infected with MA isolate of SARS-CoV-2, termed MA10, we found that BALB/c mice infected with the clinical isolate of B.1.351, a β-variant, caused lung pathology with inflammatory infiltrates that led to the induction of impaired respiratory functions (Figs1il and 2a), accompanied with the onset of a transient but significant reduction in SpO_2_ at 4 dpi (Fig. 2d). We also revealed that the presence of prominent lymphoid aggregates within the lungs of the majority of B.1.351-infecetd mice at 4 weeks (Fig. S1), a finding largely consistent with that reported by using MA10/aged (12 months) BALB/c model [28]. It is known that organized lymphoid aggregates typically develop within perivascular areas that could act as immune niches at local tissues to promote adaptive immune responses under enduring inflammatory conditions, such as infection, autoimmunity and age-related diseases, and usually disappear when the inflammation is resolved [29,31]. While the nature and cellular components of these lymphoid aggregates identified in B.1.351- or MA10-infected mice remain unknown, they might indicate the existence of ‘yet-to-be’ resolved inflammatory responses within the lungs in the post-acute phase of the disease. However, additional studies are needed in the future to better understand their impact on the pathogenesis of long-COVID.

Patients suffered from SARS-CoV-2 infection often exhibited hypercoagulable phenotype, as evidenced by activated platelets and NET, resulting in significant risks of development of severe diseases and mortality [32,34]. A sustained elevation of platelet activation and hypercoagulative responses among survivors of COVID-19, following their hospital discharge, has been reported in multiple cases [34]. Our findings revealed an increase in platelet activation in the lungs of BALB/c mice at 2 dpi, accompanied by a rise in platelet aggregates in arterioles at both 4 and 8 dpi, and persisted up to 28 dpi after B.1.351 infection with the neutrophil activation at 2 and 4 dpi. However, this platelet aggregation was not associated with lung function which may indicate a new role of platelet activation in SARS-CoV-2 infection. Further studies are required to ascertain that the induction of coagulopathy is an outcome commonly associated with B.1.351 or other variants-of concern (VOC) strains of SARS-CoV-2 infection in BALB/c mice.

The animal model serves as an invaluable tool in elucidating the mechanisms underlying neuro-PASC/neuro-COVID, especially during the post-pandemic era. To date, numerous animal models have been established for studies of SARS-CoV-2 pathogenesis and the development of effective prophylactic and therapeutic treatments. Many laboratories, including ours, have revisited the pathogenetic mechanism of SARS-CoV-2 infection using existing animal models, especially mice and hamsters, aiming to reestablish them as suitable models for long- and/or neuro-COVID. While the effort to establish such animal models capable of inducing symptoms, especially those related to dysregulated neurological functions, in response to SARS-CoV-2 infection, is still ongoing, most studies with MA10-infected BALB/c mice largely focused on delineating the pathogenesis within the lungs; very few explored the impact of respiratory SARS-CoV-2 infection on the brain/CNS [20,28]. One study using BALB/c mice infected with the MA10 virus revealed microglial activation, astrogliosis and perivascular cuffs of inflammatory infiltrates within the brain that could be only observed with aged (1-year-old) female mice at 30 dpi [35]. Several hACE2-Tg mice have been established and successfully used as models for SARS-CoV-2 infection and diseases, including K18-hACE2 and AC70-hACE2-Tg mice. We realized that the pathogenesis of SARS-CoV-2 infection derived from studies using these hACE2-Tg mice, in which hACE2 transgene was constitutively overexpressed throughout the body, including lung and brain, might not faithfully reflect that of human infections. While the effort to reestablish the AC70 hACE2-Tg mouse model for studying the pathogenesis of long- or neuro-COVID is ongoing, we also revisited an earlier claim that intranasal administration of MA10 virus to BALB/c mice largely restricted the infection within the lung, but not brains by using B.1.351 β variant, which is known to infect BALB/c mice without the need to adapt in mice [36]. While live progeny viruses could only be recovered from the lungs of B.1.351-infected BALB/c mice at 2 and 4 dpi, viral RNAs could be readily detected within the brains of most infected mice (Fig. 4a). Verification of viral replication can be achieved through the detection of SARS-CoV-2 subgenomic RNA (sgRNA). Nevertheless, the identification of low copies of genomic RNA with undetectable virus progeny in the infected BALB/c brain in this study indicated that the extent of active viral infection in the brain, if any, was exceedingly minimal and below the detection limit. Alternatively, those detected viral RNAs in the brain might be originating from the circulation through disrupted BBB or other ‘to-be-explored’ routes of the infected mice. Further studies have indicated that viral RNAs detected were nonuniformly distributed across different anatomic regions of the brain (Fig. 5a), an observation consistent with the findings using post-mortem brains of patients who succumbed to COVID-19 [37,38]. We also found that notable activation of genes encoding for *NF-kB*, *IL-6*, *IP-10* and *RANTES* starting at 2 dpi and sustained at least up to 8 dpi (Fig. 4b–e). Like the non-uniform nature of viral RNA expression, we demonstrated the levels of transcriptional activation of genes encoding inflammatory mediator cytokines/chemokines were differentially expressed over various anatomic regions of the brain (Fig. 5b–e). In addition, microglial activations were solely detected within the brains at 2, 4 and 8 dpi in B.1.351-infected mice, but not mock-infected ones (Fig. 4f–j). However, the activation of microglial processes was homogenously increased throughout the brain. Together, these results suggest that microglial activation revealed by infected mice could be induced directly upon viral infection or indirectly through the local and/or circulating inflammatory cytokines or chemokines triggered during the acute phase of viral infection. Although we have identified the presence of viral RNA in diverse anatomical regions of the brain, further investigation, e.g. RNAscope or other techniques for spatial SARS-CoV-2 gene expression within the brain tissue, is needed to ascertain the infected cell types. Additionally, the markedly elevated level of *IL-6* expression together with a high number of TRM T cells in the infected mouse brain was observed at 28 dpi following the initial infection (Fig. 6). Further investigation is necessary to determine if sustained IL-6 induction and brain TRM T cells after infection could alter the neurological functions, leading to the development of long-term neurological disorders in BALB/c mice.

In the context of the nervous system, neurons are the most structurally and functionally significant components of the brain. Modifications of gene expression and/or neuronal functions may lead to the disruption of neural circuits, which represents a pivotal pathological mechanism in the development of neurological disorders. Neurons are divided into several subpopulations and differentially distributed across brain regions [39,41]. For example, the most representative neurons in the OB are dopaminergic and the numbers of such neurons often determine the level of TH expression, a rate-limiting enzyme in catecholamine neurotransmitter synthesis [42,44]. It has been shown that BALB/c mice infected with SARS-CoV-2 N501Y_MA30_ revealed that the infected mice exhibited hypomimia/anosmia. Additionally, there was a reduction in the transcriptional and translational levels of TH at OB. Notably, neither the virus protein nor RNA could be detected within the brain of the infected mice [45]. In contrast, we showed the readily detectable viral RNAs in the OB of most mice infected with B.1.351 virus of a β variant, along with significantly down-regulated transcriptional expression of TH-encoded gene within the same region. The results indicated that at least one gene involved in neurotransmitter synthesis in OB of BALB/c mice was altered following infection with the SARS-CoV-2 B.1.351 β variant. Nevertheless, whether SARS-CoV-2 B.1.351 infection could similarly alter the transcription of other genes or neurotransmitters of different populations of neuronal cells residing at different regions of the brain remains to be elucidated.

In conclusion, the results of our study indicate that SARS-CoV-2 B.1.351 β variant infection in BALB/c mice could induce pathophysiological changes in both lung and brain comparable to a certain degree to those described for confirmed COVID-19 patients. Additional studies are ongoing to further characterize this BALB/c/β-variant of SARS-CoV-2 infection as a model that can better recapitulate symptoms reported for long- or neuro-COVID patients.

## supplementary material

10.1099/jgv.0.002039 Uncited Fig. S1.

## Abbreviations

ACE2: angiotensin-converting enzyme-2
BBB: blood–brain barrier
CNS: central nervous system
CoVs: coronaviruses
EF50: midexpiratory flow
IHC: immunohistochemistry
MERS-CoV: Middle East Respiratory Syndrome coronavirus
PASC: post-acute sequelae of COVID
Penh: enhanced pause
qPCR: quantitative PCR
Rpef: peak expiratory flow rate
SARS-CoV-2: severe acute respiratory syndrome coronavirus-2
SpO2: saturated peripheral blood oxygen
TCID50: 50% tissue culture infectious dose
UTMB: University of Texas Medical Branch, Galveston
