# Supplementary material for: BALB/c mice challenged with SARS-CoV-2 B.1.351 β variant cause pathophysiological and neurological changes within the lungs and brains
Source: J Gen Virol. 2024 Oct 30;105(10):002039. doi: 10.1099/jgv.0.002039 (PMC11524415; doi:10.1099/jgv.0.002039)
Supplement: Uncited Fig. S1. [file jgv-105-02039-s001.pdf]

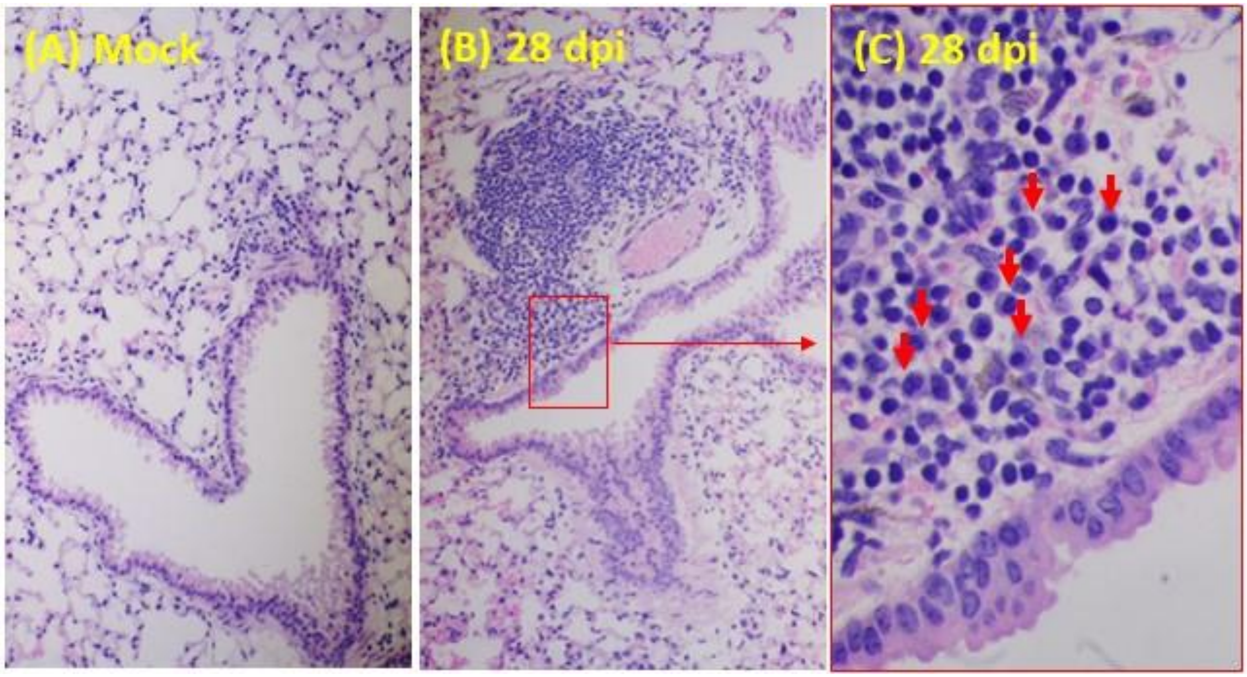

Figure S1. SARS-CoV-2 B.1.351 infection induced lymphoid aggregates in the lung of infected BALB/c mice at 28 dpi. Histology of mock-infected-lung **(A)** and SARS-CoV-2 infected lung which showed foci of lymphoid aggregates **(B)** sounding with plasma cells **(C, red arrows)**. Represent image of lymphoid aggregates from four out of five (4/5) infected mice.

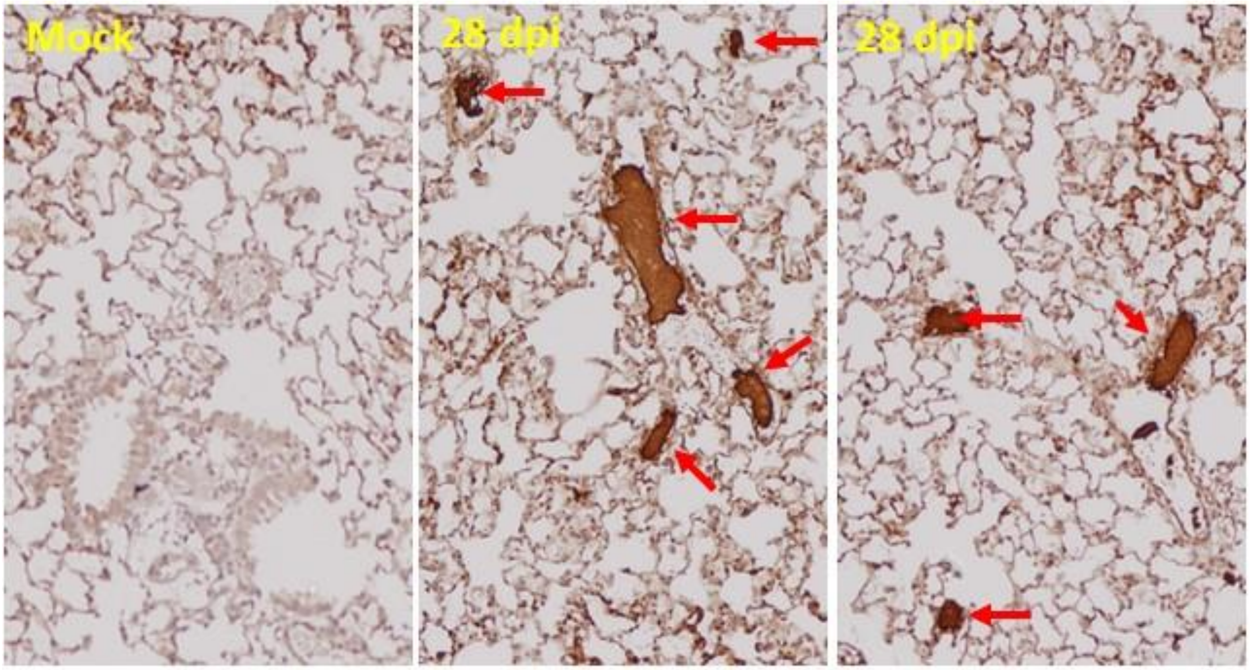

Figure S2. Platelet aggregation within arterioles at 28 dpi after SARS-CoV-2 B.3.51 infection in BALB/c mice. Representative images of immunohistochemistry stained against PF-4-antibody from a mock **(mock)** and two infected mice **(28 dpi)** out of total five mice. Mock infected mice showed some immunoreactivity while numbers of platelet aggregates were seen in arterioles of SARS-CoV-2 infected BALB/c mice **(red arrow)**.

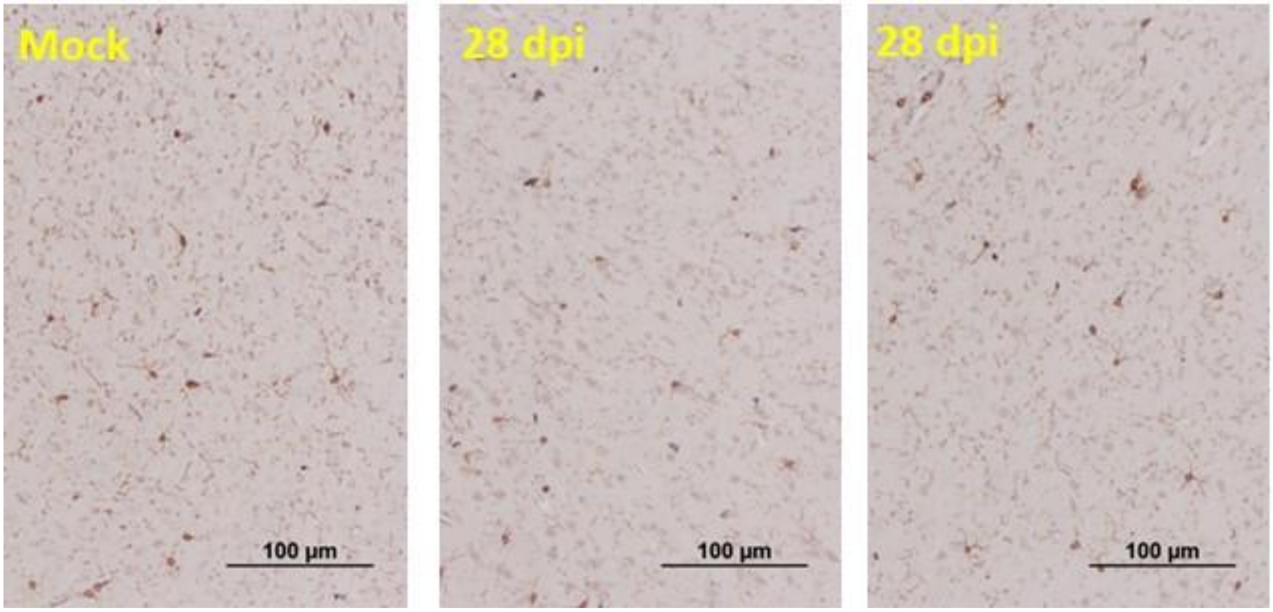

Figure S3. Microglia activation was no longer detectable within the brains at 28 dpi in SARS-CoV-2 B.1.351 infected BALB/c mice. Immunohistochemistry stained against Iba1 antibody in mock **(mock)** and two represented SARS-CoV-2 infected BALB/c mice brain **(28 dpi)**.
